# Comparison of T-cell receptor diversity of people with myalgic encephalomyelitis versus controls

**DOI:** 10.1186/s13104-023-06616-4

**Published:** 2024-01-04

**Authors:** Joshua J Dibble, Ben Ferneyhough, Matthew Roddis, Sam Millington, Michael D Fischer, Nick J Parkinson, Chris P Ponting

**Affiliations:** 1 MRC Human Genetics Unit, Institute of Genetics and Cancer, University of Edinburgh, Crewe Road South, Edinburgh, EH4 2XU UK; 2 Systems Biology Laboratory UK, Abingdon, Oxfordshire OX14 4SA UK

**Keywords:** Myalgic encephalomyelitis, T-cell receptor, Support vector machine, Targeted gDNA sequencing, CD4+ cells, CD8+ cells

## Abstract

**Objective:**

Myalgic Encephalomyelitis (ME; sometimes referred to as Chronic Fatigue Syndrome) is a chronic disease without laboratory test, detailed aetiological understanding or effective therapy. Its symptoms are diverse, but it is distinguished from other fatiguing illnesses by the experience of post-exertional malaise, the worsening of symptoms even after minor physical or mental exertion. Its frequent onset after infection suggests autoimmune involvement or that it arises from abnormal T-cell activation.

**Results:**

To test this hypothesis, we sequenced the genomic loci of α/δ, β and γ T-cell receptors (TCR) from 40 human blood samples from each of four groups: severely affected people with ME; mildly or moderately affected people with ME; people diagnosed with Multiple Sclerosis, as disease controls; and, healthy controls. Seeking to automatically classify these individuals’ samples by their TCR repertoires, we applied P-SVM, a machine learning method. However, despite working well on a simulated data set, this approach did not allow statistically significant partitioning of samples into the four subgroups. Our findings do not support the hypothesis that blood samples from people with ME frequently contain altered T-cell receptor diversity.

**Supplementary Information:**

The online version contains supplementary material available at 10.1186/s13104-023-06616-4.

## Introduction

The host immune system responds to diverse foreign antigens, partly through recombination of variable, diversity, and joining (V, D and J) chromosomal segments within T-cell receptor (TCR) α, β, $\gamma$, and $\delta$ loci in thymocytes. Random nucleotide insertion and deletion in CDR3 (complementarity-determining region 3) segment junctions provide theoretical yields of over 10^15^ different human TCR-αβ receptors [1] dispersed among approximately 10^11^ naïve T-cells circulating at any one time [2].

When T-cells are sampled from blood, most TCR sequences are observed only once, although some are found multiply in part due to identical recombination events occurring in the thymus [3] and in part due to clonal expansion of T-cells whose TCR binds to an antigen-bound major histocompatibility complex protein. Clonal expansion of T-cells occurs in disease states [4–9] and in response to infection, for example with Epstein-Barr virus [10]. A minority (~ 10%) of individuals with acute viral or bacterial infection experience a prolonged illness lasting 12 months [11, 12]. Many people with myalgic encephalomyelitis (ME) report an onset of symptoms after infection [13–15]. ME is a multi-systemic disabling disease that results in a health-related quality of life worse than 20 other conditions including chronic inflammatory disorders, cancer and multiple sclerosis [16, 17]. A quarter of people with ME report being house- or bed-bound [18]. ME is not rare: it affects, for example, between 836,000 and 2.5 million Americans [16] and an estimated 0.2–0.4% of the UK population [19, 20]. Despite its high prevalence and burden of illness, no reliable biomarker or diagnostic test exists and its aetiology is unknown.

Given that its onset is frequently ascribed to infection and hypotheses that it may be an autoimmune condition [16] we set out to determine whether blood samples from people with ME show an expansion of TCR clonotypes compared with healthy or disease controls.

## Results

### Samples and entropy metrics

Forty human peripheral blood mononuclear cell (PBMC) samples were received from each of four groups: (i) Severely affected people with ME (either house- or bed-bound; MEsa); (ii) Mildly or moderately affected people with ME (MEmm); (iii) people diagnosed with Multiple Sclerosis (MS; disease controls); and, (iv) Healthy controls (HC). Samples were sourced from the CureME Biobank [21] from female donors aged 40–60 years, chosen to reduce possible age- or sex-dependent confounding effects, although their limited availability among MEsa required us to source samples from younger donors (Table 1).

**Table 1:** PBMC samples

Age at sample collection	18–29 years	30–39 years	40–49 years	50–60 years
Severely affected people with ME, MEsa	8	9	6	17
Mildly- or moderately-affected people with ME, MEmm	0	0	20	20
People with Multiple Sclerosis, MS	0	0	12	28
Healthy controls, HC	0	1	16	23

Six CD8^+^ samples were discarded: one had insufficient enriched cells, five libraries had been cross-contaminated. Among CD4^+^ samples, 12 were discarded: two had insufficient enriched cells, four had insufficient DNA, and six failed sequence library synthesis, leaving 154 CD8^+^ samples and 148 CD4^+^ samples. Analysis of variance (ANOVA) for the experimental parameters (Table 2) showed that the only association with sample group was CD8^+^ cell number$(p=0.03$). This was not significant after accounting for multiple tests.

**Table 2:** Mean values of cell count and purity, and numbers of CD8^+^ or CD4^+^ samples

	PBMC Input				CD8^+^ Enriched output						CD4^+^ Enriched output			
	Sample No.	PBMC cells (x${10}^{6}$)	CD8^+^ (%)	CD4^+^ (%)	Sample No.		CD8^+^ cells (x${10}^{6}$)	CD8^+^ (%)	CD4^+^ (%)		Sample No.	CD4^+^ cells (x${10}^{6}$)	CD4^+^ (%)	CD8^+^ (%)
MEsa	40	5.27	15.7	29.0	38		0.632	70.7	2.6		35	0.665	72.0	1.4
MEmm	40	4.50	15.1	30.2	38		0.541	70.3	3.2		37	0.703	76.0	1.7
MS	40	4.62	14.2	34.6	39		0.473	68.0	3.6		39	0.652	73.5	1.4
HC	40	4.72	16.3	30.6	39		0.585	69.8	2.7		37	0.698	73.6	1.3
All	160	4.78	15.3	31.1	154		0.558	69.7	3.1		148	0.679	73.7	1.4

Human herpesvirus infection may be a trigger for ME [22], and Cytomegalovirus (CMV) also affects T-cell clonal diversity [23, 24]. CMV infection is very common in the UK [25] although CMV seropositivity in this study, available for 98 of the 160 samples, was not significantly associated with sample group status ($p=0.58$, χ^2^-test; Table 3).

**Table 3:** Cytomegalovirus seropositivity/seronegativity results, shown by group. Equivocal samples were those with indeterminate IgG levels

Seropositivity	MEsa	MEmm	MS	HC
Positive	14	4	11	12
Negative	19	9	14	13
Equivocal	2	0	0	0

We chose Rényi entropy as our T-cell receptor diversity metric (Additional file 1). For each cell type and α/β/γ-chain combination we defined a vector of clonotype counts in a sample. Here, clonotype is defined as CDR3, plus the full V, D and J gene segments without considering α−β or γ−δ chain pairing. Note that $\delta$-chain data is excluded, because recombination of the $\alpha$ locus, which occurs first, preferentially removes the TCR-δ locus [26]. Next, we constructed a matrix of Euclidean distances between vector pairs, ensuring that each pair contained equivalent numbers of recombinant rearrangements by randomly down-sampling the more-populous sample (Additional file 1), a necessity as TCR rearrangement counts varied over two orders of magnitude among samples. Distances were calculated over a pre-set optimised range of α, the order of Rényi entropy. Next, cell type and α/β/γ-chain combinations were partitioned by clonotype, adapting a machine learning approach [27] (Additional file 1). Once distances were precomputed, investigators were unblinded to the group identity (e.g. MEmm) of each CD8^+^ sample. CD4^+^ data were acquired following unblinding.

### Multidimensional scaling (MDS) plots

**Fig. 1:**
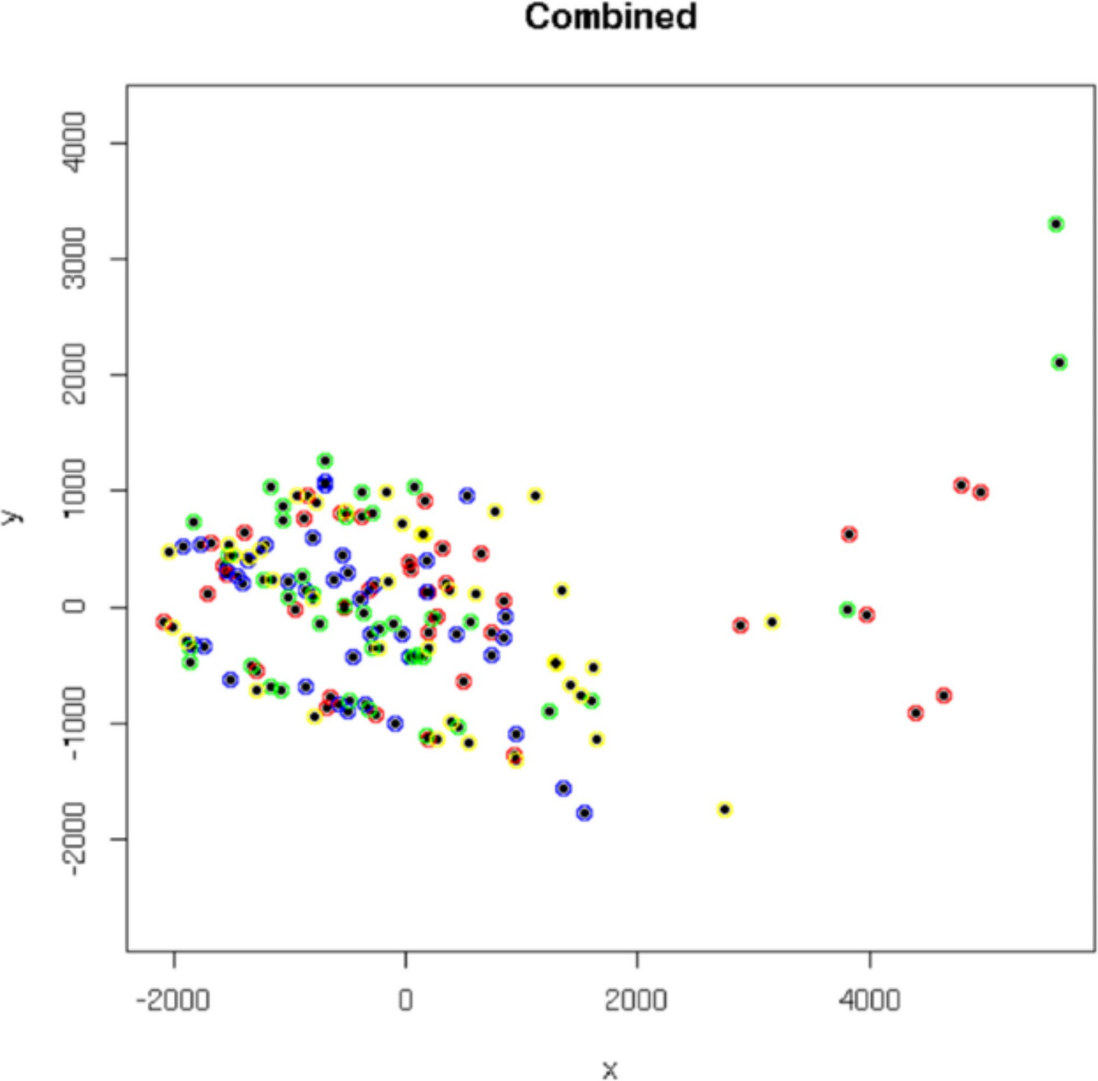
Multidimensional scaling (MDS) plot of all CD8 + chains. Samples from MEsa are indicated in red, MEmm in yellow, MS in green and HC in blue. Axes indicate projected distances, such that the linear distance between two samples reflects the value in the distance matrix. There is no clear visual separation between the groups in two dimensions, but there are notable disease case (MEsa, MEmm and MS) outliers for $x>\text{2,000}$. The plot was visualised using *cmdscale()* in *R*. MDS plots are invariant under rotation and reflection

The distance matrix for all three CD8^+^ chain types (TCR α-, β-, and γ-chains) considered together was visualised using a MDS plot (Fig. 1). The four groups (MEsa, MEmm, MS and HC) are not clearly separated in this plot’s two dimensions. Location of seven MEsa, two MEmm and three MS disease cases away from the main cluster ($x>2000$) are likely due to age, rather than disease status, because among 21 variables (Table 4), only age (binned by decade) was strongly negatively and significantly correlated with *x*-axis values ($p=2\times {10}^{-3}$). This likely reflects a narrowing of immune repertoire diversity due to age-associated reduced thymic activity (thymopoiesis) [28], which also explains our finding that age group is a significant predictor ($p = 8\times {10}^{-3}$) of CD8^+^ TREC count. Four of seven of the MEsa outliers (Fig. 1) were from individuals aged 18-29 years at donation.

**Table 4:** Variables tested as potential confounders with MDS plot x-axis using a generalised linear model fit

Variable	Estimate	Std. Error	t-value	$\varvec{P}\varvec{r}(>|\varvec{t}\left|\right)$	Significance
(Intercept)	1.25E + 11	2.22E + 11	0.564	0.574	
ME(mm)	1.89E + 02	3.20E + 02	0.590	0.556	
ME(sa)	4.51E + 01	3.68E + 02	0.123	0.903	
MS	4.73E + 01	3.23E + 02	0.147	0.884	
Age	-1.36E + 03	4.29E + 02	-3.16	0.00199	**
PBMC Number	1.35E + 02	1.02E + 02	1.32	0.190	
CD8+ % Input	-4.54E + 01	2.87E + 01	-1.58	0.116	
CD8 + Cells Post-selection	-7.90E + 02	7.85E + 02	-1.01	0.316	
Purity	6.47E + 00	8.34E + 00	0.776	0.439	
DNA Extracted (µg)	-9.18E + 01	1.51E + 02	-0.608	0.544	
DNA Yield (ng)	6.11E-03	3.90E-01	0.016	0.988	
Demultiplexed Reads (641)	1.23E-04	5.47E-05	2.25	0.0263	*
PEAR Assembled (641)	1.19E + 01	1.69E + 01	0.701	0.485	
Demultiplexed Reads (782)	-1.56E-05	4.27E-05	-0.366	0.715	
PEAR Assembled (782)	-1.39E + 01	1.87E + 01	-0.741	0.460	
α Coding Junctions	5.74E-01	2.38E-01	2.41	0.0174	*
β Coding Junctions	-4.18E-01	3.32E-01	-1.26	0.211	
γ Coding Junctions	-8.88E-01	3.07E-01	-2.90	0.00452	**
α %	-1.25E + 11	2.22E + 11	-0.564	0.574	
β %	-1.25E + 11	2.22E + 11	-0.564	0.574	
γ %	-1.25E + 11	2.22E + 11	-0.564	0.574	

Significance levels for the t-test statistic are shown: for $p<0.001$ (***), $p<0.01$ (**), and $p<0.05$ (*). ME(sa), ME(mm) and MS were testing whether being a member of the given subgroup was correlated with the axis, relative to healthy controls (HC). Age (binned by decade) was the linear term of the model t for that covariate. Other covariates: PBMC Number, total number of cells per sample; CD8^+^% Input, percentage of input PBMCs that were CD8^+^ cells; CD8^+^ cells post-selection, number of CD8^+^ cells after the SureSelect process; Purity, corresponding CD8^+^ purity at this stage; DNA Extracted (µg), total amount of DNA initially recovered from each sample; DNA Yield (ng), amount remaining after size selection; Demultiplexed Reads [Library], total number of reads recovered for the two Illumina flowcells of sufficient quality (library numbers 641 and 782); PEAR Assembled [Library], percentages assembled into clonotypes for the same libraries using the Paired-End Read Merger tool [37]; α/β/γ Coding Junctions, total numbers of coding junctions for each chain type; α/β/γ %, percentages of each chain type, relative to the other two types (such that the sum was 100%)

**Fig. 2:**
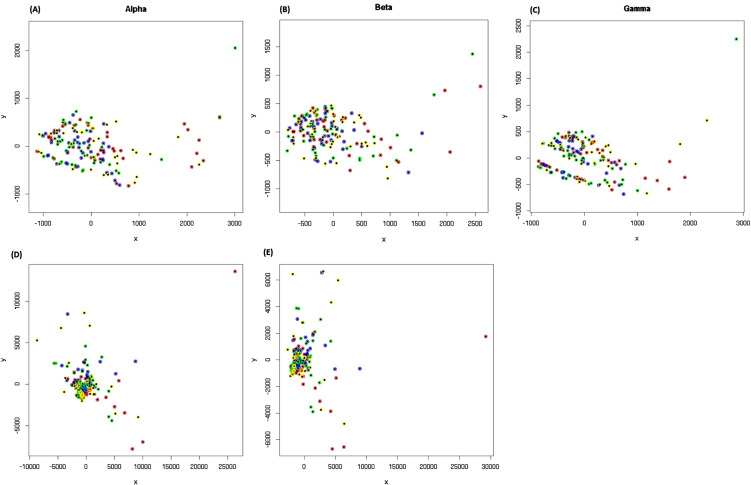
Multidimensional scaling plots for the three TCR chain types of CD8 + cells. Panels **A**, **B**, **C**: Data from α, β and γ chains are shown. Samples from MEsa are indicated in red, MEmm in yellow, MS in green and HC in blue. No clear separation by group is visible in two dimensions. Panels **D** and **E**: MDS plots for α-chain CD8^+^ data showing changes to the distance matrix caused by adjusting the α-step size (Panel D, $\alpha$-range of $\left[\text{0,20}\right]$ and a step size of 0.1) and $\alpha$-range (Panel E, $\alpha$-range of $[0,,10]$ and a step size of 0.2, as in [27])

**Fig. 3:**
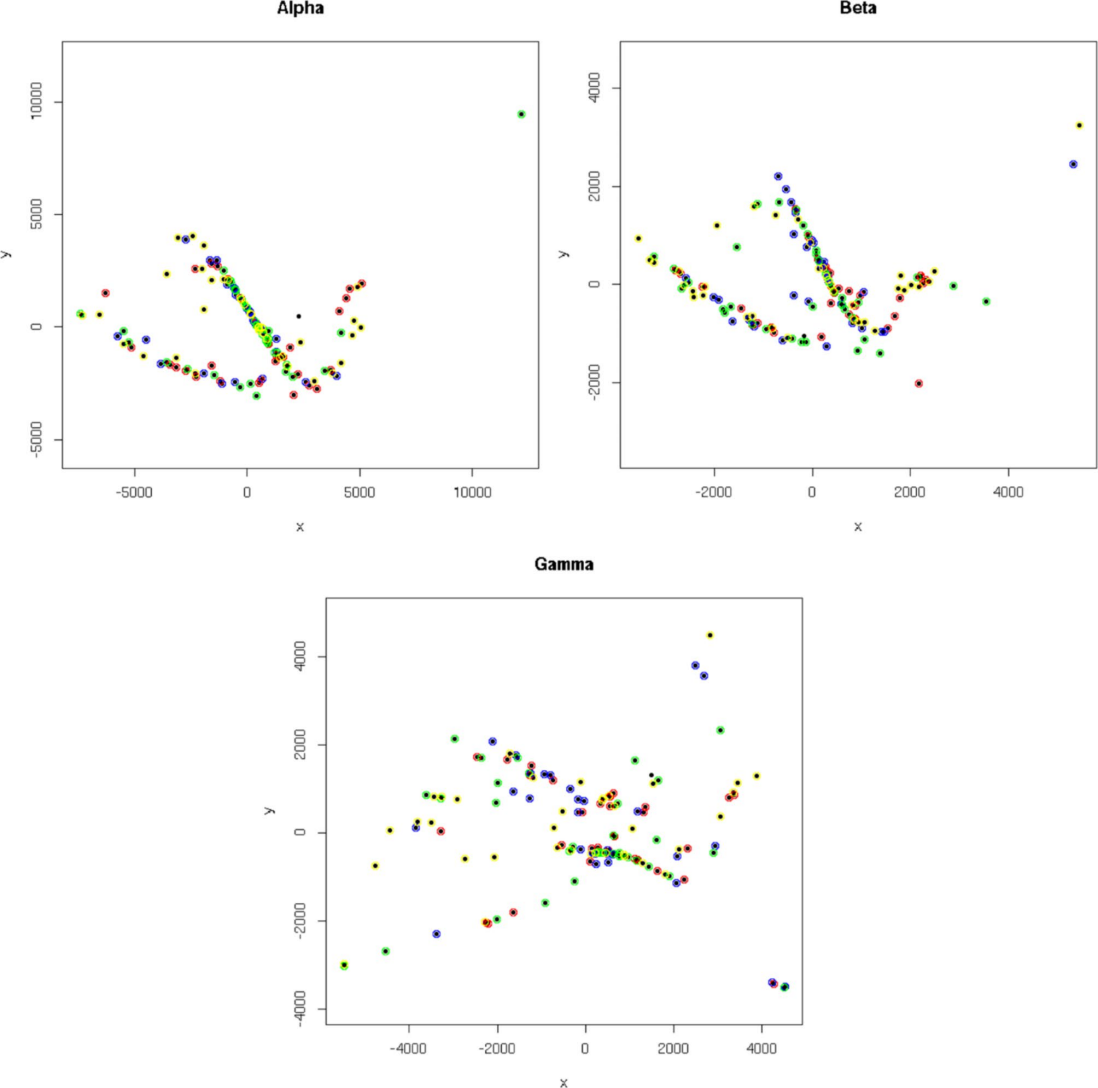
Multidimensional scaling plots for the three TCR chain types of CD4 + cells. α, β and γ chain data analyses are shown clockwise from the top left. Samples from MEsa are indicated in red, MEmm in yellow, MS in green and HC in blue. No clear separation by group is visible in two dimensions

MDS y-axis values were negatively correlated with β (*TRB*) locus coding junction count ($p=1\times {10}^{-3}$; Table 5) motivating us to perform similar analyses that separated CD8+ rearrangements by each of the three loci: TCR α-, β- or γ-loci. Again, no clear separation was visible in two dimensions (Fig. 2A,B,C), or when a third dimension was added, or when different α-ranges and step size were used (Fig. 2D,E). Finally, we undertook this analysis for CD4^+^ (helper T) cells, separating by TCR α-, β-, or γ-chain. Although some structure was apparent, again we observed no clear separation between groups in two dimensions (Fig. 3).

**Table 5:** Variables tested as potential confounders with MDS plot y-axis using a generalised linear model fit

Variable	Estimate	Std. Error	t-value	$\varvec{P}\varvec{r}(>|\varvec{t}\left|\right)$	Significance
(Intercept)	4.93E + 10	8.91E + 10	0.553	0.581	
ME(mm)	-1.48E + 01	1.29E + 02	-0.115	0.908	
ME(sa)	1.77E + 02	1.48E + 02	1.20	0.235	
MS	1.50E + 02	1.30E + 02	1.16	0.249	
Age	3.49E + 01	1.72E + 02	0.202	0.840	
PBMC Number	-2.28E + 01	4.11E + 01	-0.554	0.580	
CD8+ % Input	2.70E + 00	1.15E + 01	0.234	0.815	
CD8 + Cells Post-selection	3.02E + 02	3.15E + 02	0.959	0.340	
Purity	-4.29E + 00	3.35E + 00	-1.28	0.203	
DNA Extracted (µg)	2.42E + 00	6.07E + 01	0.04	0.968	
DNA Yield (ng)	1.88E-01	1.57E-01	1.20	0.232	
Demultiplexed Reads (641)	5.19E-05	2.20E-05	2.36	0.0199	*
PEAR Assembled (641)	-5.77E + 00	6.79E + 00	-0.85	0.397	
Demultiplexed Reads (782)	-6.02E-06	1.72E-05	-0.351	0.726	
PEAR Assembled (782)	-1.04E + 00	7.51E + 00	-0.138	0.890	
α Coding Junctions	1.56E-01	9.56E-02	1.63	0.105	
β Coding Junctions	-4.50E-01	1.33E-01	-3.38	0.000998	***
γ Coding Junctions	-2.47E-01	1.23E-01	-2.00	0.0474	*
α %	-4.93E + 10	8.91E + 10	-0.553	0.581	
β %	-4.93E + 10	8.91E + 10	-0.553	0.581	
γ %	-4.93E + 10	8.91E + 10	-0.553	0.581	

Significance levels are shown for $p<0.001$ (***), $p<0.01$ (**), and $p<0.05$ (*). Parameters are as for Table 4

### Potential support vector machine (P-SVM)

**Fig. 4:**
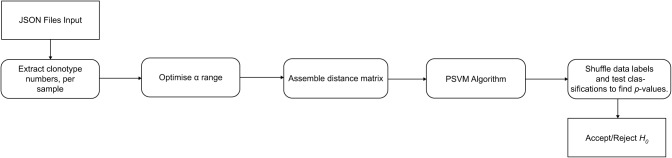
Analysis pipeline for the computational implementation of the TCR clonotype diversity analysis

We next assessed if clonotypes of one group (e.g. ME cases) clustered separately from another (e.g. healthy controls) using the Potential Support Vector Machine (P-SVM) approach [29] with leave-one-out cross-validation. Classification boundaries between groups were defined by the P-SVM and classifications were tested against random expectation using permutation testing (up to ${10}^{3}$ label shuffles; Additional file 1; Fig. 4).

Our three primary aims were to test for a difference in the expansion of T-cell clonotypes inferred from TCR sequencing between: (i) ME cases (MEsa + MEmm) and healthy controls, or (ii) ME cases and MS controls, or (iii) MEsa and MEmm cases. Recognition that each hypothesis was tested six times (both CD4^+^ and CD8^+^ cells; and each of α-, β- or γ-chains) resulted in a Bonferroni multiple testing correction [30] of $p<\frac{0.05}{18}\approx 0.0028$ for the 18 primary tests. Our six secondary aims were to test for T-cell clonotype expansion differences between: healthy controls and (a) ME or (b) MEmm or (c) MEsa cases; or between MS cases and (d) healthy controls or (e) MEmm or (f) MEsa cases for each of the two cell types and three TCR chains, i.e. $6\times 2\times 3=36$ tests. Accounting for all 54 tests, our Bonferroni-corrected threshold for testing secondary aims was $p<\frac{0.05}{54}\approx 9.3\times {10}^{-4}$.

Comparison of group classifications against random permutations yielded single-test *p*-values, each representing the likelihood that two groups had been separated by the P-SVM algorithm by chance. Four comparisons achieved significance at $p<0.05$ (Table 6). Nevertheless, after appropriate Bonferroni correction no comparison remained significant.

We conclude that none of the null hypotheses that we investigated should be rejected.

**Table 6:** Uncorrected *p*-values from permutation testing for CD4^+^ and CD8^+^ cells, by α/β/γ chain type. *p*-values $<0.05$ are indicated in bold. The † symbol indicates that fewer than 1,000 permutations were used to generate the *p*-value due to computational resource constraints

		CD8^+^			CD4^+^		
		α	β	γ	α	β	γ
**Primary Hypotheses**	ME vs. HC	0.224	1.00	1.00	0.137	**0.030**	0.053
	ME vs. MS	0.236	1.00	1.00	0.663	0.351	0.293
	MEsa vs. MEmm	0.395	0.224	0.433	0.396	0.875	0.735
**Secondary Hypotheses**	Cases vs. HC	1.00†	1.00†	1.00†	0.301†	0.104†	**0.024†**
	MS vs. HC	0.998	0.715	0.709	0.379	0.827	0.140
	MEmm vs. HC	0.547	0.207	0.301	0.454	0.385	0.231
	MEsa vs. HC	**0.020**	0.597	0.133	0.064	0.193	0.318
	MEmm vs. MS	0.193	0.451	0.553	0.135	0.491	0.514
	MEsa vs. MS	0.129	0.597	0.267	0.662	**0.020**	0.588

## Discussion

We applied a method that determines statistical significance of the difference in TCR clonotype repertoires between cases and controls. With simulated data, the approach perfectly distinguished sample groups at a 1% *p*-value threshold (Additional file 1). Nevertheless, with experimentally-determined data the method detected no statistically significant differences between TCR clonotype diversities of people with ME and others with MS or healthy control individuals, or between the TCR clonotypes of severely affected versus mildly- or moderately-affected people with ME (MEsa and MEmm). Despite this study comparing a relatively large number of samples from people with ME ($n=80$) with those from healthy controls ($n=40$) or from disease controls (MS; $n=40$), and despite a large number of rearrangements ($\sim{10}^{3}-{10}^{4}$) being sequenced from each sample, no differences between groups were detected.

Future work could consider refining the analytical pipeline using samples from a disease with a more clearly established incidence of clonal expansion such as T-cell leukaemia/lymphoma [31]. A future study might apply the Berger-Parker index which is weighted towards greater α-values; in our study low α-values gave unstable estimates. It is likely that all current TCR repertoire studies are severely underpowered at present, due to current technology greatly undersampling the true T-cell repertoire [32].

### Limitations

First, given its diverse initiating onsets and symptoms, ME may yet be found to have heterogeneous biomarkers, each predictive of only some patients. Without such biomarkers, research studies of this size may lack adequate power to identify molecular signatures, such as TCR repertoires, that are predictive of only a minority of patients. Discovery of biomarkers or clinical tests that aid ME diagnosis remains an urgent priority for future research. Second, a larger study restricted to individuals with particular HLA alleles may have greater predictive power, because *HLA* risk alleles modulate autoimmunity risk by increasing the frequency of autoreactive TCRs [33].

Third, even if ME is aetiologically homogeneous, our negative results could reflect a lack of statistical power to identify true differences. If sampling noise in our experiment’s clonotype counts obscured a clear signal, rearrangement numbers may need to be increased by several orders of magnitude, from ${10}^{4}- {10}^{5}$ sequences per donor investigated here, towards the $\sim{10}^{11}$ naïve T-cells in human repertoires [2]. Additional predictive power could be gained by investigating the pairing of TCR chains (e.g. α−β) in single cells, although this would be at the inevitable expense of greater experimental cost.

Finally, this study’s results could also reflect an absence of clonotype diversity differences between groups. If so, then the TCR clonotype sequences themselves, rather than their diversity, could be predictive of disease status, or else TCR repertoire differences are manifest not in blood, but in other more disease-relevant tissues as in MS [34]. Our negative results could also reflect causal mechanisms of ME that do not result in T-cell repertoire change.

## Materials and methods

### Donor sample cell enrichment

160 samples of peripheral blood mononuclear cells (PBMC) were acquired from the CureME Biobank [21] from the same number of anonymised donors. A unique pseudo-anonymised identifier was assigned to each sample to allow the study to be blinded. Samples were stored at -180^o^C. CureME defines ME samples according to either or both of the Canadian Consensus and Fukuda criteria [21]. Four groups of 40 samples were provided: (i) Severely affected people with ME (either house- or bed-bound; MEsa); (ii) Mildly or moderately-affected people with ME (MEmm); (iii) people diagnosed with Multiple Sclerosis (MS; disease controls); and, (iv) Healthy controls (HC). As much as possible, these groups were age-matched, with the majority of donors being between 40 and 60 years old at the time of sample collection; all were from female donors (Table 1). Samples’ CMV seropositivity status was provided by the CureME Biobank based on the level of CMV immunoglobulin G (IgG) measured in their plasma [21]. Data generation and analysis protocols were developed and optimised using two CD8^+^ samples from healthy controls (American Type Culture Collection, Manassas, VA, USA).

PBMCs were enriched by Magnetic Activated Cell Sorting [35] using MACS Micro beads (Miltenyi Biotec) first to positively select CD8^+^ (cytotoxic) cells (CD8 MicroBeads, human 130-045-201), then CD4^+^ (helper/regulatory) cells (CD4 MicroBeads, human 130-045-101). After each selection step, material was retained for verification by Flow Cytometry staining using fluorescent conjugated antibodies specific for enriched populations (Miltenyi Biotec MACS 130-113-125 CD3 Antibody, anti-human, APC Clone: BW264/56, MACS 130-113-254 CD4 Antibody, anti-human, PE Clone: M-T466, MACS 130-113-157 CD8 Antibody, anti-human, FITC Clone: BW135/80). Enrichment results were visualised with plots of co-receptor expression using manually set gates to define discrete cell populations (Additional file 5). A minor batch effect was noted, but variability was more pronounced between individuals than between batches, so was considered to have minimal impact.

### Sequencing library synthesis

Samples with > 1 × 10^5^ enriched (CD8^+^ or CD4^+^) cells were lysed and DNA extracted using a QIAmp DNA Micro Kit (Qiagen). Samples returning > 1 µg DNA were fragmented by sonication with a BioRuptor (Diagenode) using 30 rounds at 20 s intervals, size-separated using gel electrophoresis and fragments between 250 and 350 base pairs excised and recovered using a Clean Gel DNA Recovery kit (Zymo). Yield was measured using a Quant-IT Picogreen DNA assay kit (Thermo Fisher Scientific). Fragments were polished to produce blunt ends using a NextNEB End Repair kit (New England Biolabs).

Custom designed UMI adapters were ligated to polished gDNA fragments (Integrated DNA Technologies). dsDNA adapted design incorporated, in order from the ligatable blunt end: (i) a 4 base-pair (bp) validation barcode, (ii) a 6 bp random Unique Molecular Identifier (UMI), (iii) a 4 bp library ID barcode, (iv) a 2 bp *AcuI* target cleavage site, (v) a 14 bp filler sequence, (vi) a 6 bp *AcuI* binding site, (vii) a 5 thymine, 2 uridine, 5 thymine single base run forming a closed hairpin cap preventing adapter concatemerisation. Following adapter ligation (New England Biolabs), Axyprep Solid Phase Reversible Immobilisation (SPRI) beads were used to size select and clean-up the DNA (Corning). The hair-pinned adapter ends were then opened with a NEB USER kit (NEB) to enable five rounds of enrichment PCR with a Kappa Hifi Polymerase $2\times$ Mastermix kit (Roche). Amplicon yields were measured with PicoGreen (Thermo Fisher Scientific) and re-size selected by gel electrophoresis.

### Target enrichment and sequencing

A target enrichment strategy was used to select library fragments containing signal and coding recombinations of the human T-cell receptor loci on chromosomes 14q11 (*TRAD*, α/δ locus), 7q34 (*TRB*, β locus) and 7p14 (*TRG*, γ locus). Biotin-conjugated 120mer ssDNA SureSelect enrichment baits were custom designed (Agilent SureSelect) to give $2\times$ tiling depth across a 500 bp region centred on each predicted recombination signal sequence (RSS) breakpoint motif (IMGT/GENE DB v3.1.30, https://www.imgt.org; [36]). Baits for V regions (1,680) and J regions (996) were synthesised as separate libraries. Each library also contained 17 common baits designed across a 1 kb section of the 14q11 α/δ locus *TRAC* constant region with $2\times$ tiling depth for process quality control and assessment of input library coverage (see Additional file 2). The bait capture library selecting for fragments with J region homology was employed initially to enrich samples batched into 1.5 µg pools of five previously UMI indexed sample libraries, 300ng per donor, using manufacturer’s standard protocols (Agilent). The output of each J region selection was amplified with five rounds of PCR using Kappa Hifi Polymerase $2\times$ Mastermix (Roche). To enrich for recombinant library fragments containing both V and J elements this product was then used as input for a second round of selection with the V region bait library using the manufacturer’s standard protocols. Final capture products were amplified with ten rounds of PCR using Kappa Hifi Polymerase $2\times$ Mastermix (Roche).

Enriched libraries were prepared for Illumina NovaSeq sequencing. *AcuI* (NEB) was first used to digest the post-selection amplification products to remove the majority of the custom adapter whilst retaining the 4 bp validation sequence, 6 bp UMI, 4 bp library ID barcode and a predictable, 2 bp “sticky” overhang. Custom designed barcoded Illumina compatible adapters with complementary 2 bp overhangs were then ligated to each library pool (IDT). Final library pools were enriched using five cycles of PCR, size selected by gel electrophoresis and sequenced on an Illumina NovaSeq platform using a $2\times$ 250 bp PE sequencing protocol (Arizona Genetics Core).

### Data analysis

A custom suite of Java programmes was used for library demultiplexing and assigning clonotypes. Read pairs were quality and length filtered (all bases with Phred $>20$, >250 bp) then demultiplexed using both Illumina pool indexes and library specific barcodes to identify high quality donor-specific subsets of reads. Successfully validated 6 bp UMI sequences were identified from each read in a pair, concatenated to form a fragment specific 12 bp UMI sequence, recorded in the ID field of each read along with barcoding information. These sequences were then trimmed from the fragment ends and reads collapsed to make a library of $1\times$ 250 bp SE QC filtered, UMI annotated and trimmed reads for each donor.

A bioinformatics library of 20 base unique sequence tags was curated to unambiguously identify each V, D or J element of the human T-cell receptor loci on chromosomes 14q11 (*TRAD*, α/δ locus), 7q34 (*TRB*, β locus) and 7p14 (*TRG*, γ locus). Tags were positioned 15 bp from the predicted element RSS site and reference sequences recorded (Additional file 3). For tags predicted to span known single nucleotide variants multiple alternative versions were produced to permit allelic discrimination. Where local homology prevented unique tag production, non-unique tags (rtags) shared by multiple elements were generated. Secondary discriminating tags (alt-tags) located at the nearest point of divergence were then used to differentiate between rtag elements during processing. Separate libraries of tags were produced for ‘coding’ and ‘signal’ flanks of each RSS. Libraries of donor specific $1\times$ 250 bp SE reads were scanned bioinformatically to identify reads with homology to tags in pairwise conformations compatible with VDJ signal or coding recombination. Parallel comparison of VDJ junction identification was conducted using PEAR [37] pre-assembly of overlapping 250 bp PE reads to reconstitute, where possible, the full-length sequenced fragment.

CDR3 regions were defined for such reads as nucleotide sequences located between the predicted position of identified V, D or J element RSS cleavage sites or where homology to the expected reference approaching the RSS site diverged. Individual V-CDR3-J cassettes were determined to be productive or non-productive by scanning for a single ORF containing the known coding frames of each V or J element identified. Predicted in-frame stop codons were also noted and only productive rearrangements were analysed further. Each unique V-CDR3-J sequence was assumed to represent a discrete T-cell clonotype and sequencing reads with identical V-CDR3-J recombination were clustered within each donor library. PCR derived duplicates were identified and removed from these clusters using degenerate matching of fragment 12 bp UMI sequences, allowing for up to two base mismatches. Clonotype duplicates within a donor library with unique UMI sequences were assumed to represent T-cell clonal expansions. The number of unique clonotypes and extent of their expansion was then scored for each donor library to give an index of T-cell diversity.

### Generalized linear models (GLMs)

To investigate MDS axes we implemented a generalized linear model using *glm()* in R (see Additional file 1).

### Electronic supplementary material

Below is the link to the electronic supplementary material.

Additional File 1: T-cell Receptor Diversity Estimation

Additional File 2: All SureSelect bait sequences used

Additional File 3: Human T-cell Receptor loci recombination signal sequence (RSS) and Tag sequences used to unambiguously identify each V, D or J element

Additional File 4: Table providing sample metadata and information

Additional File 5: S Fig. 1 Example Flow Cytometry analysis of enriched cells populations

## Data Availability

The CD4^+^ and CD8^+^ sequencing reads datasets generated during and/or analysed during the current study are available in the Sequence Read Archive repository, https://www.ncbi.nlm.nih.gov/sra: SAMN33477947-SAMN33478250 (304 files). Software used to analyse this project’s data, alongside instructions and all reference sequence tags, can be found on. https://gitlab.com/SBLUKcic/vdj-recombine. Project data and meta-data are available as Additional file 4.
