# Supplementary material for: Comparison of T-cell receptor diversity of people with myalgic encephalomyelitis versus controls
Source: BMC Res Notes. 2024 Jan 4;17:17. doi: 10.1186/s13104-023-06616-4 (PMC10768444; doi:10.1186/s13104-023-06616-4)
Supplement: Supplementary file 1 — Additional File 1: T-cell Receptor Diversity Estimation [file 13104_2023_6616_MOESM1_ESM.docx]

**Additional information** for Comparison of T-cell Receptor Diversity of people with Myalgic Encephalomyelitis versus controls by Dibble, Ferneyhough, Roddis et al.

*T-cell Receptor Diversity Estimation*

Many metrics used for estimating the diversity of the human T-cell repertoire are specific instances of a more general measure, the Rényi entropy [1]. Although Shannon entropy [2] and Simpson's diversity [3] are commonly used in the T-cell literature, others argue that metrics are often applied uncritically, without considering what is being measured [4]. To avoid biasing results by choosing a specific diversity metric prior to analysis, we decided to use the more general, and thus more appropriate, Rényi entropy (**Methods**).

The Rényi Entropy of order α is:

$$H_{\alpha}=\frac{1}{1-\alpha}\log\sum_{i=1}^{s} p_{i}^{\alpha}$$

where $\alpha$ is the weighting parameter and $p_{i}$ is the frequency of the $i^{th}$*^h^* category:

$$p_{i}=\frac{x_{i}}{\sum_{i} x_{i}}$$

and the input is a vector of counts for a sample $x$: $x=(x_{1}, x_{2}, x_{3}, \ldots, x_{s})$. Taking the exponent of the Rényi entropy for input vector $x$ results in a diversity index profile $D_{\alpha}=e^{H_{\alpha}}$ for a given array of α-values. Choice of $\alpha$ yields different TCR diversity profiles: higher values of $\alpha$ increase the weight assigned to the most frequently occurring clonotypes, whereas when $\alpha=0$ all clonotypes are weighted equally, irrespective of how many instances of each are found. To define a generalised distance between each pair of TCR diversity vectors we used their Euclidean distance calculated over a range of $\alpha$ ($\alpha_{min}\leq\alpha\leq\alpha_{max}$, step 0.1).

The range of $\alpha$-values was optimised before unblinding using a zero-point calibration. For this we first selected a sample $i$, and drew a set of 9 random sub-samples of recombinants, at 10% size intervals, from the original set (90%, 80%, etc) which, together with the original data sample, made a set of 10 (sub-)samples. For each sample pair $(i, j)$, we calculated the Euclidean distance (as above) between pairs of their 10 (sub-)samples, summing over different $\alpha$-interval ranges $[X,Y]$, where $X<Y$, with step size 0.5. The $\alpha$-range that yielded the minimum non-zero median distance between the two samples $(i, j)$ was then considered to be optimal. Based on these results $\alpha$-values were chosen for CD8^+^ cells ($30\leq\alpha\leq50$) reflecting the mean values of the lower and upper bounds of 27.1 and 47.1, respectively. An $\alpha$-value step-size of 0.1 was chosen, providing twice the resolution used by Greiff et al. [5]. For consistency, the same parameters were later used for the CD4^+^ cell data.

Using sequenced data from two CD8^+^ healthy control samples, we found that Rényi entropy estimates are not robust to subsampling, in contrast to previous claims [5]. To account for the wide variation in samples’ recombinant counts, we adopted a subsampling approach. For each sample pair $[a,b]$ (with sizes $a>b$) we randomly subsampled $a$ to the size of $b$, repeatedly on $n$ occasions, before calculating the Euclidean distance between $b$ and each $a$ subsample and then taking the median over the $n$ values to define their diversity profiles $D_{\alpha}$. Results from the two CD8^+^ healthy control samples led us to choose the number of resamples, $n=1,000$.

*Classification using the Potential Support Vector Machine (P-SVM)*

We considered various types of non-parametric supervised algorithms to separate cases and controls into groups by the distances calculated between diversity profiles [6]. We chose to apply a SVM approach as it is a distance-based algorithm that draws a classification boundary between two labelled sets of data, and as it accepts a kernel function to allow non-linear boundaries [6-9]. Further, we chose to implement the P-SVM approach of Hochreiter *et al.* [9], a modified SVM which was formulated to work on pairwise relationship data, such as distance matrices [9]. This has been shown to typically outperform similar SVM approaches [9], and has the added advantage of having been tested previously on T-cell clonotype counts data [5].

We used the P-SVM to attempt to accurately classify the clonotype repertoires into the four groups: severe ME/CFS, mild/moderate ME/CFS, multiple sclerosis, and healthy controls. The P-SVM handles multiple classes iteratively, finding one classification boundary at a time, using leave-one-out cross-validation (rather than training and testing datasets), with optimised hyperparameters [9, 10]. For this step, a single data point is removed, the model is trained on the remaining data, and then the model predicts the status of the removed data point. This process is repeated for each data point in turn. Another leave-one-out loop is used to select the optimal model hyperparameters, $\varepsilon$ and $C$, where $\varepsilon$ is the level of tolerance for errors in the training data (if too high the model will fail to capture nuance in the training set, and if it too small it will overfit) and $C$ controls the maximum weighting of the support vectors. If left at the default value ($C=1$) then there are few support vectors, leaving the model more prone to outliers. Setting $C$ too low normally provides a less accurate classification boundary [10]. Optimisation of these two hyperparameters was achieved by minimising the generalization error, here the mean squared error [10].

Significance testing was achieved by randomly shuffling data labels (up to 1,000 times) and then training the machine on these shuffled datasets. This generated a distribution of accuracy scores (sum of true positive and true negative classifications). Statistical significance is achieved at the 5% level when fewer than 5% of scores from the shuffled datasets exceed the original model’s accuracy score. An advantage of this implementation of the P-SVM is that the hyperparameters are set per individual permutation. Whilst this dramatically increases the computation time, it is more robust to false positives, because applying the hyperparameters from the initial data for all permutations will lead to a better fit on the actual data than on the permutations. Applying the hyperparameter values acquired from the optimal fit to real data to permutations was found to yield an erroneous significant result.

*Simulation*

To demonstrate that the P-SVM could differentiate between clonotype repertoires with or without signs of expansion, we created a simulated data set of 160 samples, each with 10^4^ recombinants. Half were ‘severe cases’, whose recombinant counts were drawn from power law distributions with exponents -1.5 (40 samples) or -1.4 (40 samples), 40 were ‘mild/moderate cases’ using exponent -0.2, and 40 were ‘healthy controls’ whose counts were drawn from a uniform distribution. Applying the P-SVM workflow described above (using 100 resamples, $30\leq\alpha\leq50$ and $\alpha$ step-size of 1) resulted in perfect discrimination of sample labels at a 1% significance threshold.

1. Renyi A: **On Measures of Entropy and Information.** *Proceedings of the Fourth Berkeley Symposium on Mathematical Statistics and Probability* 1961, **1:**547-561.

2. Shannon CE: **A Mathematical Theory of Communication.** *The Bell System Technical Journal* 1948, **27:**379-423.

3. Simpson EH: **Measurement of Diversity.** *Nature* 1949, **163:**688.

4. Alatalo RV: **Problems in the Measurement of Evenness in Ecology** *Oikos* 1981, **37:**199-204.

5. Greiff V, Bhat P, Cook SC, Menzel U, Kang W, Reddy ST: **A bioinformatic framework for immune repertoire diversity profiling enables detection of immunological status.** *Genome Med* 2015, **7:**49.

6. Rashidi HH, Tran NK, Betts EV, Howell LP, Green R: **Artificial Intelligence and Machine Learning in Pathology: The Present Landscape of Supervised Methods.** *Acad Pathol* 2019, **6:**2374289519873088.

7. Bzdok D, Krzywinski M, Altman N: **Machine learning: supervised methods.** *Nat Methods* 2018, **15:**5-6.

8. Noble WS: **What is a support vector machine?** *Nat Biotechnol* 2006, **24:**1565-1567.

9. Hochreiter S, Obermayer K: **Support vector machines for dyadic data.** *Neural Comput* 2006, **18:**1472-1510.

10. **Potential - Support Vector Machine (P-SVM) website** [**https://www.tu.berlin/en/ni/research/software-and-data/psvm**](https://www.tu.berlin/en/ni/research/software-and-data/psvm)
