# Supplementary material for: Comparison of T-cell receptor diversity of people with myalgic encephalomyelitis versus controls
Source: BMC Res Notes. 2024 Jan 4;17:17. doi: 10.1186/s13104-023-06616-4 (PMC10768444; doi:10.1186/s13104-023-06616-4)
Supplement: Supplementary file 5 — Additional File 5: S Fig. 1 Example Flow Cytometry analysis of enriched cells populations [file 13104_2023_6616_MOESM5_ESM.docx]

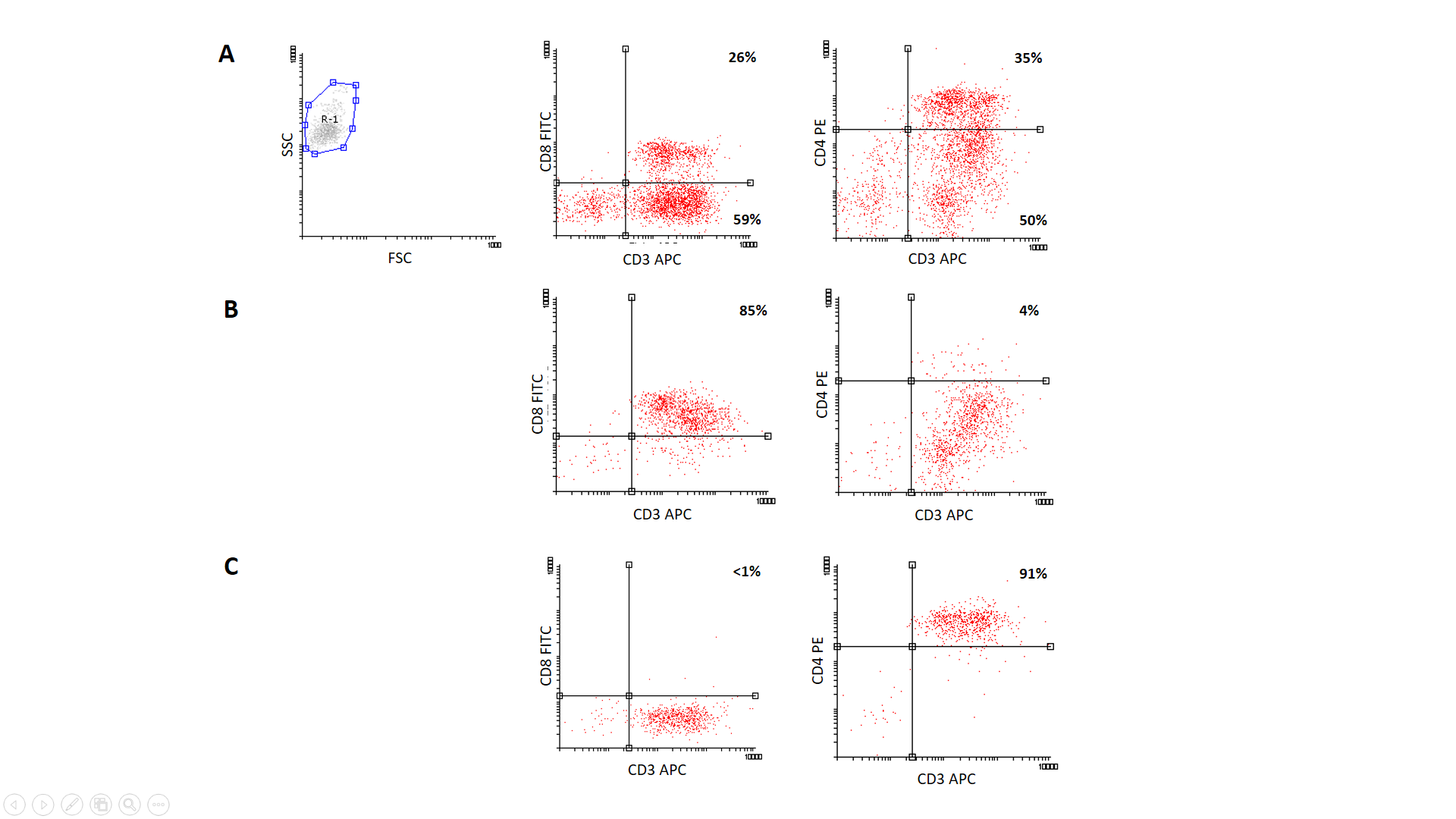


**S Figure 1. Example Flow Cytometry analysis of enriched cells populations.**

The composition of PBMCs (A) and subsequently enriched CD8^+^ (B) and CD4^+^ (C) cell populations were ascertained by Flow Cytometry staining using fluorescent conjugated antibodies specific to cell surface T cell markers (Miltenyi Biotec CD3-APC, CD8-FITC, CD4-PE).
